# Characterizing homozygosity across United States, New Zealand and Australian Jersey cow and bull populations

**DOI:** 10.1186/s12864-015-1352-4

**Published:** 2015-03-15

**Authors:** Jeremy T Howard, Christian Maltecca, Mekonnen Haile-Mariam, Ben J Hayes, Jennie E Pryce

**Affiliations:** Department of Animal Science, North Carolina State University, Raleigh, NC 27695-7627 USA; Dairy Futures Cooperative Research Centre, 5 Ring Road, Bundoora, Victoria 3083 Australia; La Trobe University, Bundoora, Victoria 3086 Australia; Biosciences Research Division, Department of Environment and Primary Industries Victoria, 5 Ring Road, Bundoora, 3083 Australia

**Keywords:** Dairy cattle, Runs of homozygosity, Signature of selection

## Abstract

**Background:**

Dairy cattle breeding objectives are in general similar across countries, but environment and management conditions may vary, giving rise to slightly different selection pressures applied to a given trait. This potentially leads to different selection pressures to loci across the genome that, if large enough, may give rise to differential regions with high levels of homozygosity. The objective of this study was to characterize differences and similarities in the location and frequency of homozygosity related measures of Jersey dairy cows and bulls from the United States (US), Australia (AU) and New Zealand (NZ).

**Results:**

The populations consisted of a subset of genotyped Jersey cows born in US (n = 1047) and AU (n = 886) and Jersey bulls progeny tested from the US (n = 736), AU (n = 306) and NZ (n = 768). Differences and similarities across populations were characterized using a principal component analysis (PCA) and a run of homozygosity (ROH) statistic (ROH45), which counts the frequency of a single nucleotide polymorphism (SNP) being in a ROH of at least 45 SNP. Regions that exhibited high frequencies of ROH45 and those that had significantly different ROH45 frequencies between populations were investigated for their association with milk yield traits. Within sex, the PCA revealed slight differentiation between the populations, with the greatest occurring between the US and NZ bulls. Regions with high levels of ROH45 for all populations were detected on BTA3 and BTA7 while several other regions differed in ROH45 frequency across populations, the largest number occurring for the US and NZ bull contrast. In addition, multiple regions with different ROH45 frequencies across populations were found to be associated with milk yield traits.

**Conclusion:**

Multiple regions exhibited differential ROH45 across AU, NZ and US cow and bull populations, an interpretation is that locations of the genome are undergoing differential directional selection. Two regions on BTA3 and BTA7 had high ROH45 frequencies across all populations and will be investigated further to determine the gene(s) undergoing directional selection.

**Electronic supplementary material:**

The online version of this article (doi:10.1186/s12864-015-1352-4) contains supplementary material, which is available to authorized users.

## Background

The widespread use of dense single-nucleotide polymorphism (SNP) assays for genomic prediction has led to the creation of large reference populations across multiple countries and breeds [1,2]. Previous studies have utilized these assays to identify and characterize regions of the genome that have undergone positive selection, referred to as selection signatures [3-9]. Selection signatures are characterized by distributions of nucleotides around favorable mutations that differ statistically from that expected purely by chance due to directional selection increasing the frequency of the favorable allele over time [10]. Nucleotides linked to the favorable mutation also tend to increase in frequency a phenomenon referred to as “hitchhiking” [11]. A recent study by Kemper et al. [9] provided evidence that locating signatures of selection is difficult for complex traits due to hundreds of loci associated with the trait undergoing weak selection. Even though a selection signature is difficult to detect for complex traits, selection does change the allele frequency of loci associated with the trait. Turchin et al. [12] showed that specific alleles of SNP associated with human height were at a higher frequency in northern than southern Europe, which mirrors observations of differences in height in European populations.

A potential alternative to detect signatures of selection for complex traits may be to characterize regions of the genome that have a higher likelihood of occurring within a continuous run of homozygosity (ROH) [13,14]. A ROH is generated when an individual receives a haplotype that is identical by descent from each parent [14]. Furthermore, parents can pass on identical chromosomal segments to a child even when the relationship between them is a very distant one, which creates a continuum of homozygous length, depending on the degree of shared ancestry and its age [15]. In dairy cattle the use of artificial insemination has allowed elite bulls to produce thousands of progeny, resulting in a high frequency of familial relationships within the pedigree, potentially giving rise to a high and non-uniform distribution of ROH frequency across the genome within a given population. Utilizing a ROH based metric, referred to as locus autozygosity, on United States (US) Holstein sires, Kim et al. [8] showed that differences in the location and distribution of ROH regions varied across groups that underwent different degrees of selection pressure. Furthermore, multiple regions that they declared as different were found to be associated with milk yield traits.

In general dairy cattle breeding programs have similar breeding objectives, regardless of country, that are driven by traits of economic importance such as milk, fat and protein yield along with fertility, longevity and conformation. The environments and management conditions in which individual animals perform differ greatly across countries. This is confirmed by genetic correlations varying from 0.75 to 0.80 between US and New Zealand (NZ) and US and Australia (AU) for milk, fat and protein yield [16]. Furthermore, the relative importance of a given trait varies across countries potentially giving rise to different selection pressures across the genome. Selection in North America is mainly practiced in environments where confinement and total mixed ration are typical management settings, in comparison to NZ and AU where performance is predominantly in pasture based systems. Different management systems may lead to variation in the importance of a given genomic region, thereby differentially increasing the frequency of favorable alleles. For example, Kolver et al. [17] confirmed that North American Holstein-Friesian cows have a greater capacity to convert feed to milk when fed a total mixed ration type diet in comparison to cows from NZ. Studies involving Holsteins have confirmed that the NZ Holstein is genetically different than Holstein derived from other European and North American countries [18-20]. Recently Pryce et al. [18] combined genotype panels on Holstein animals from multiple research herds (North America, Europe, AU, NZ) and conducted a PCA analysis on the genomic relationship matrix (GRM) and found slight differences across research herds with the greatest difference arising in the NZ population compared to the other research herds.

A limited number of studies have investigated genetic differences across countries within the Jersey breed [21]. Characterizing what causes these subtle changes at the genomic level within the Jersey dairy cattle breed is worthwhile because of the higher levels of inbreeding and smaller effective population size in Jerseys when compared to the Holstein breed [22,23]. Furthermore, lower correlations of production and fertility traits evaluated in northern (US) and southern (NZ and AU) hemisphere countries have been estimated for the Jersey breed in comparison to the Holstein breed [24], which could make detection of regions of the genome that are under differential selection across countries more insightful. Also, in comparison to the Holstein breed, there has been somewhat less international gene exchange, therefore characterizing differences across population could allow for more efficient collaborations to enhance genomic improvement. The objective of this study was to characterize differences and similarities in the location and frequency of homozygosity related measures of Jersey dairy cows and bulls from the United States US, AU and NZ.

## Results

### Population stratification and average homozygosity across the genome

The populations utilized to make comparisons across populations consisted of a subset of genotyped Jersey cows born in the US (n = 1047) and AU (n = 886) and Jersey progeny tested bulls from the US (n = 736), AU (n = 306) and NZ (n = 768). The SNP in common across the 2 cow and 3 bull populations totaled 31,431 and 27,927, respectively. Each sex was analyzed separately as different selection pressures are likely to exist across sexes and a higher level of diversity is expected within the cow populations. The number of animals within a year on the complete set is outlined in Table 1. To assess the degree of differentiation across populations, a principal component analysis (PCA) on the genomic relationship matrix constructed separately for the cows and bulls based on Yang et al. [25] was used. Also, a traditional measure of population differentiation, Wrights F_st_ statistic, was computed as outlined by Weir & Cockerham [26] and reported as the average F_st_ of a moving 8 SNP window. Measures used to characterize the homozygosity averaged across the genome included the proportion of the genome that is homozygous and the proportion of the genome that is contained within a ROH. Using a sliding window approach, a ROH was declared when a region of 45, 70 or 95 contiguous homozygous SNP with no heterozygotes was observed.

**Table 1:** **Number of animals by birth year within each population** ^**1**^ **for cows and bulls**

	**Cows** ^**2**^		**Bulls** ^**2**^		
	**AU**	**US**	**AU**	**NZ**	**US**
1990-1994	2	106	85	174	325
1995-1997	18	61	101	213	271
1998-2000	202	26	132	327	326
2001-2003	871	137	155	403	324
2004-2006	2009	1121	151	365	412
2007-2009	886	3076	90	74	420
≥ 2010	3	3672	175	0	53

^1^AU = Australia; US = United States; NZ = New Zealand.

^2^Principle component analyses and characterizing the autozygosity between and within populations used animal born within years 2007 to 2009 and 2001 to 2006 for the cows and bulls, respectively. Change in autozygosity across time used cows born after 2002 for the US population and AU cows born from 1990 and 2010. Change in autozygosity across time used bulls born between 1999 to 2008 for AU, NZ, and US.

**Figure 1:**
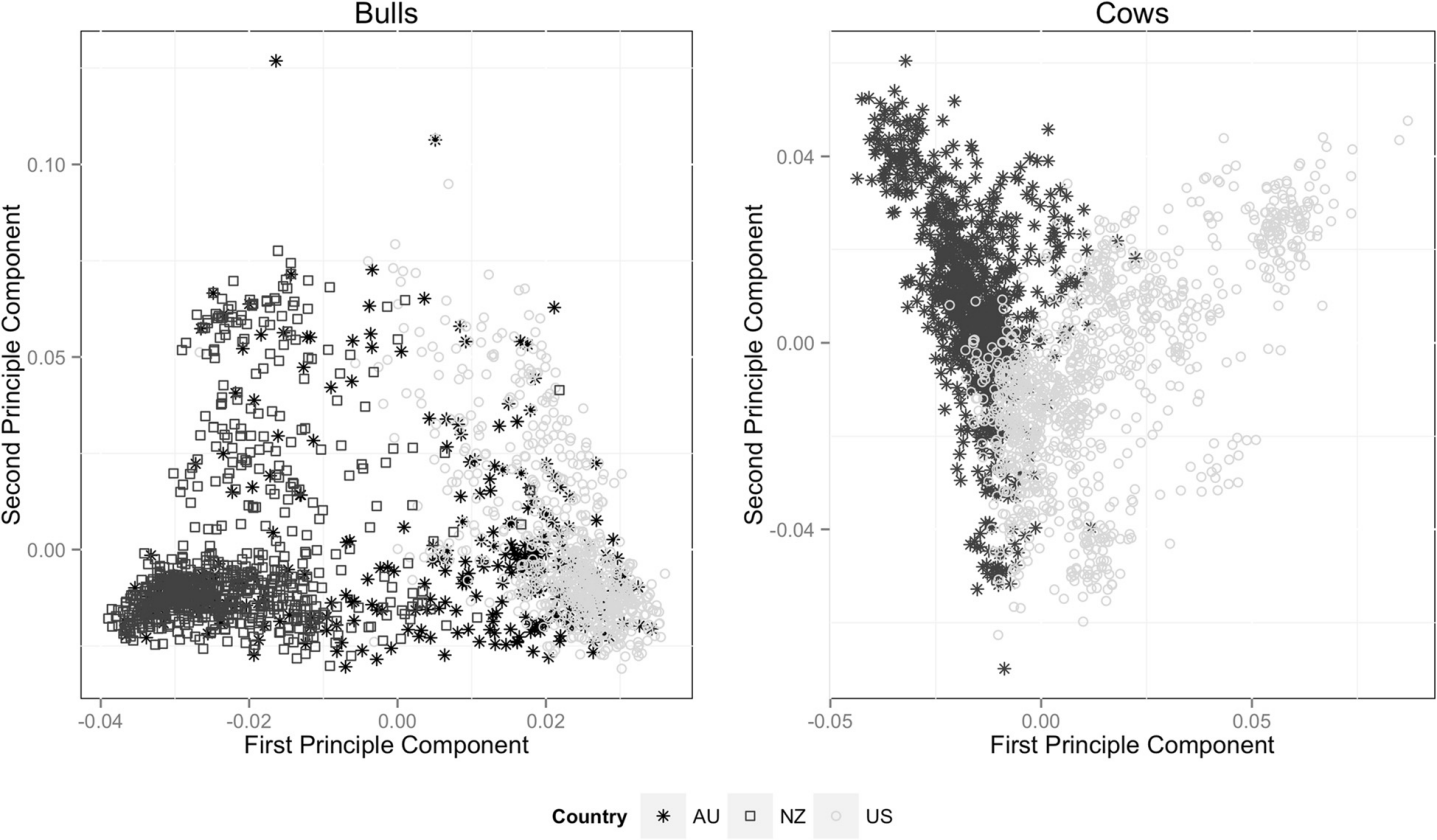
**First vs Second PC analysis for the bull** ^**1**^ **and cow** ^**2**^ **analysis by population.** ^1^Number of bulls in the analysis was 1810: 736 from United States (US); 306 from Australia (AU); 768 from New Zealand (NZ). ^2^Number of cows in the analysis was 1933: 1047 from US; 886 from AU.

Figure 1 is a scatterplot of the first principal component (PC1) of the GRM versus the second principal component (PC2). The US and NZ bulls show some degree of differentiation and the variance explained by PC1 is 0.056 percent. Furthermore the AU bulls appear to be a mix of the NZ and US population, although within the AU population some bulls, as judged by their genotypes, are more similar to US than NZ and vice versa. Differentiation between the AU and US cow population is marginal in comparison to the bulls and the variance explained by the first PC is 0.024. The mean (max) F_ST_ across the genome for AU versus US cows was 0.008 (0.12) and the average (max) for US versus AU, US versus NZ, and AU versus NZ was 0.006 (0.08), 0.029 (0.21) and 0.009 (0.07), respectively. Although some differentiation based on F_st_ was seen, especially for US versus NZ bulls, the other populations appear to be similar. The average (±SD) homozygosity for each metric is outlined in Table 2. The US cow and bull populations have higher levels of homozygosity across all four of the metrics in comparison to the AU cow population and AU and NZ bull population. The NZ bull population has the lowest levels of homozygosity in comparison to the AU and US bull population.

**Table 2:** **Average (±SD) ROH homozygosity by population** ^**1**^

	**Cow (2007–2009)**		**Bull (2001–2006)**		
**Metric**	**AU (n = 886)**	**US (n = 1047)**	**AU (n = 306)**	**US (n = 736)**	**NZ (n = 768)**
ROH_45_	0.130 (0.038)	0.139 (0.042)	0.129 (0.045)	0.148 (0.043)	0.099 (0.031)
ROH_70_	0.099 (0.038)	0.108 (0.041)	0.100 (0.043)	0.117 (0.042)	0.071 (0.030)
ROH_95_	0.078 (0.036)	0.087 (0.039)	0.080 (0.041)	0.097 (0.040)	0.054 (0.029)
Proportion Homozygous	0.667 (0.014)	0.666 (0.017)	0.669 (0.018)	0.672 (0.017)	0.658 (0.014)

^1^AU = Australia; US = United States; NZ = New Zealand.

### Characterizing the frequency of autozygosity across and within populations

**Figure 2:**
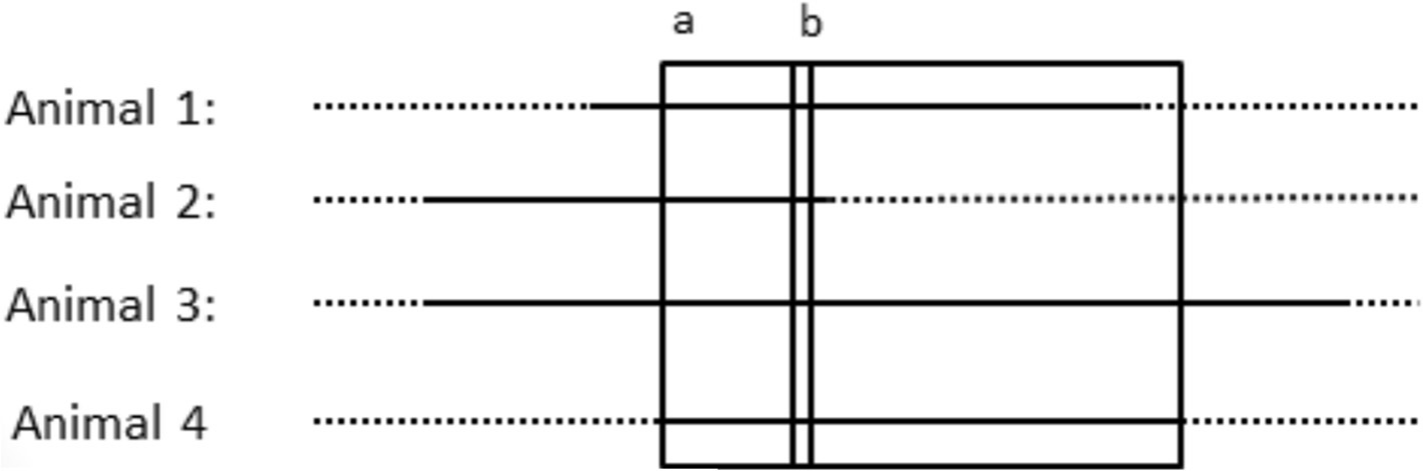
**The local autozygosity (ROH45)** ^**b**^ **metric in contrast to the traditional ROH metric, where the solid lines represent a ROH region and dashed lines represent a region not in an ROH.** The ROH45 metric is able to capture animals with slightly different start and stop sites for a particular ROH region, which is illustrated by animal 1 versus animal 3 and 4. ^a^refers to the large box and is the traditional ROH metric. ^b^refers to the small box and is the ROH status of a SNP (ROH45).

In order to detect subtle differences across the genome in the location and length of long stretches of homozygosity, the ROH metric of SNP length 45, described by Kim et al. [8], was calculated. The ROH45 metric has advantages over conventional ROH because it captures the number of times a SNP is in a ROH without declaring the beginning and end of a ROH. Therefore, animals with slightly different start and stop sites for a particular ROH region will still be grouped into a SNP that is in a ROH region which is illustrated graphically in Figure 2. The ROH45 value for each SNP was compared across the two populations using a chi-square test and a statistical threshold determined using a permutation test [27]. A region was declared different across populations when at least 45 contiguous significant (P-value < 0.001) SNP were detected in a region greater than 4 Mb. Regions of high ROH45 frequency (top 2.5%) that were at least 45 contiguous SNP and greater than 4 Mb across all populations were considered similar across populations.

**Figure 3:**
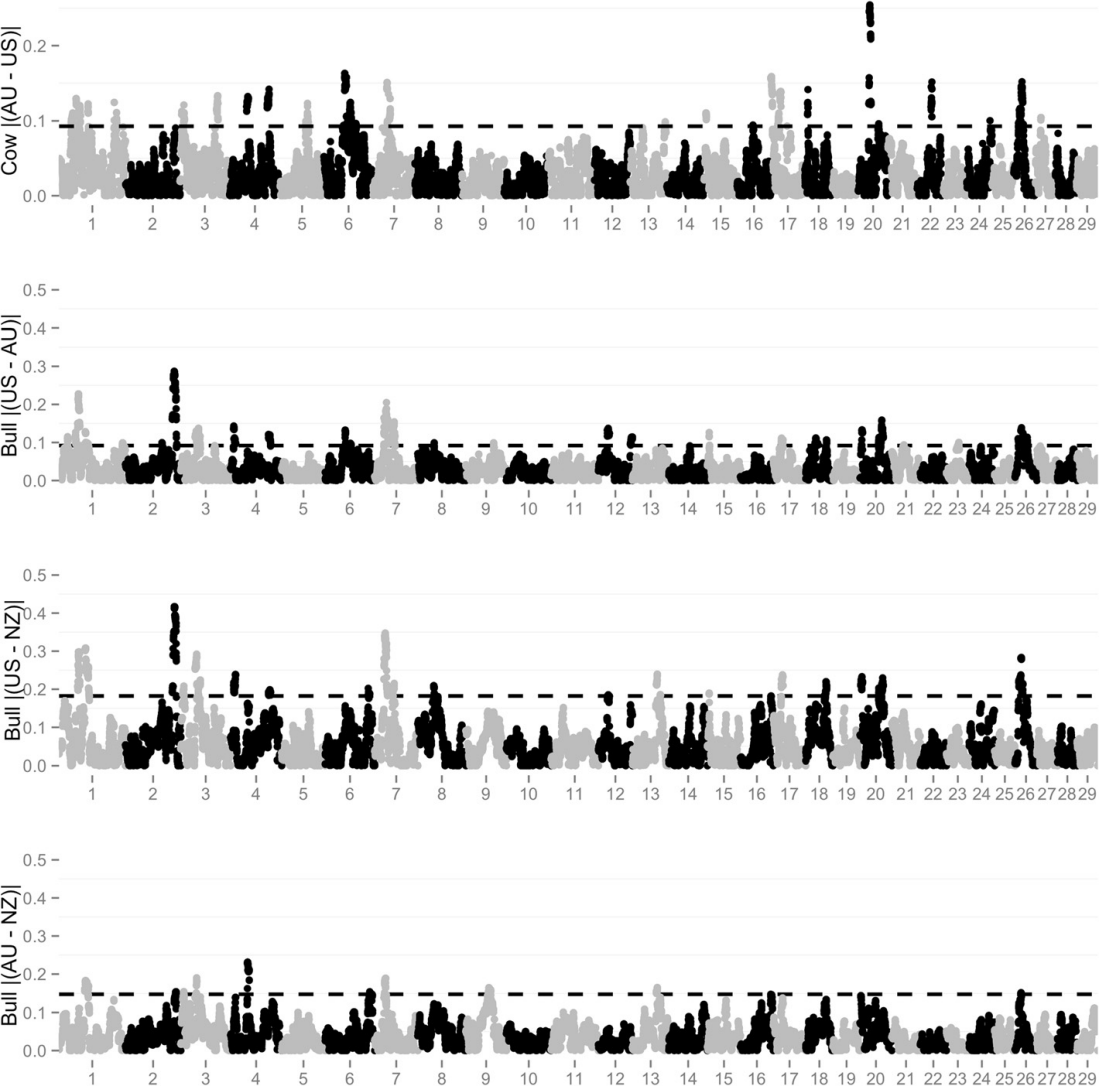
**Autozygosity within each bull** ^**1**^ **and cow** ^**2**^ **population.** ^1^Number of bulls totaled 1810: 736 from United States (US); 306 from Australia (AU); 768 from New Zealand (NZ). ^2^Number of cows totaled 1933: 1047 from US; 886 from AU. ^3^Dashed line represents top 2.5% of autozygosity values.

**Figure 4:**
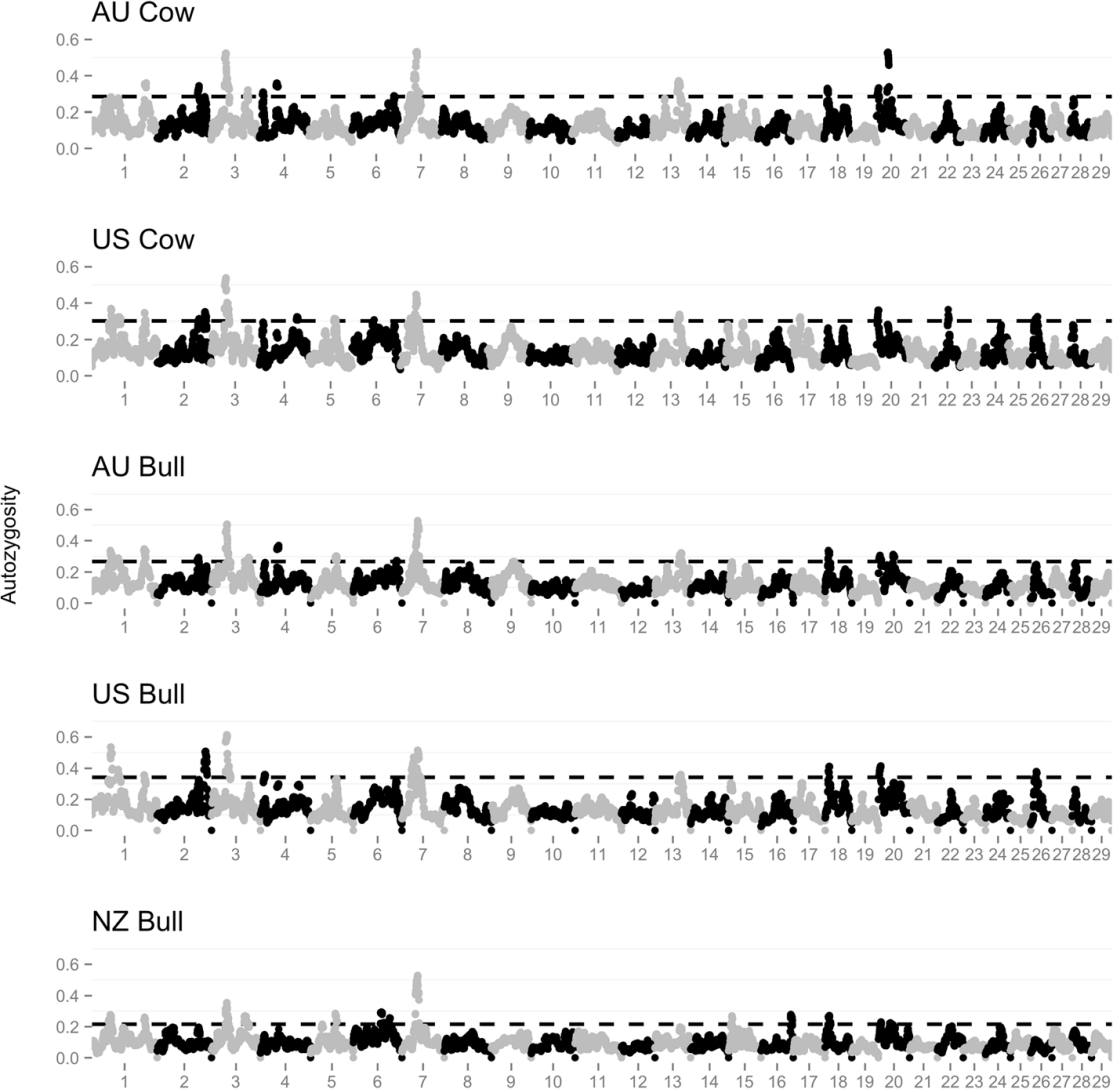
**Pairwise absolute difference across bull** ^**1**^ **and cow** ^**2**^ **populations in autozygosity across the genome.** ^1^Number of bulls totaled 1810: 736 from United States (US); 306 from Australia (AU); 768 from New Zealand (NZ). ^2^Number of cows totaled 1933: 1047 from US; 886 from AU. ^3^Dashed line represents significance threshold (P-value < 0.001).

Regions with high levels of autozygosity for all populations were detected on BTA3 (38.8 to 55.2 Mb) and BTA7 (35.6 to 48.9 Mb), as shown in Figure 3. Across all populations the location of the maximum ROH45 frequency for BTA3 and BTA7 was between 42.4 - 44.2 and 35.6 and 41.4 Mb, respectively. Multiple regions of the genome displayed different autozygosity frequencies as outlined in Table 3 and displayed graphically in Figure 4. The number of regions that were different across populations was greatest for the US and NZ bull comparison, which is in agreement with the difference between the two populations obtained by the principal component analysis and by the F_ST_ metric.

**Table 3:** **Regions of the genome that have different ROH45 frequencies across bull** ^**1**^ **and cow** ^**2**^ **populations**

		**ROH45 Difference (Maximum Location** ^**4**^ **)**			
**Chr** ^**3**^	**Largest Interval** ^**4**^	**US vs AU Cows**	**US vs AU Bulls**	**US vs NZ Bulls**	**AU vs NZ Bulls**
1	6.5 -27.1	-	-	0.17 (14.4)	0.10 (18.6)
1	48.9-55.4	-	-	0.30 (51.3)	-
1	61.2-77.9	-	-	0.31 (67.3)	0.18 (67.3)
2	80.2-94.1	-	-	0.17 (89.1)	-
2	119.2-129.4	-	0.28 (123.9)	0.42 (125.2	0.15 (128.4)
3	1.7-10.5	-	-	0.21 (6.6)	0.16 (6.6)
3	39.1-46.0	-	-	0.29 (43.9)	0.19 (43.9)
3	46.7-62.0	-	-	0.22 (57.1)	0.14 (57.1)
4	8.0-12.2	-	-	0.24 (11.7)	-
4	41.2-46.4	0.13 (45.0)	-	0.16 (41.7)	0.23 (41.7)
4	80.7-86.7	-	-	0.14 (84.5)	-
4	92.1-96.4	0.14 (96.3)	-	0.20 (94.8)	-
4	102.2-106.2	-	-	0.15 (103.4)	0.13 (101.6)
6	47.2-51.8	0.16 (47.8)	-	-	-
6	55.6-61.1	-	-	0.14 (57.0)	
6	102.2-106.2	-	-	0.20 (103.2)	0.15 (105.6)
7	19.9-34.6	-	-	0.35 (27.1)	0.19 (28.0)
7	49.0-58.4	-	-	0.22 (56.1)	-
8	20.4-24.8	-	-	0.13 (21.6)	
8	37.9-51.7	-	-	0.21 (41.6)	0.12 (50.7)
8	52.1-57.4	-	-	0.15 (53.2)	-
9	57.0-74.3	-	-	0.14 (60.1)	0.17 (62.3)
9	86.8-92.3	-	-	0.12 (88.7)	-
11	18.7-24.4	-	-	0.15 (21.9)	-
12	22.9-29.4	-	-	0.19 (27.3)	-
12	85.8-91.1	-	-	0.16 (86.7)	-
13	59.3-67.3	-	-	0.24 (61.7)	-
14	72.4-79.8	-	-	0.15 (75.6)	0.12 (75.0)
16	51.9-60.0	-	-	0.16 (56.3)	-
17	.1-4.2	0.16 (2.2)	-	-	-
17	12.6-25.2	-	-	0.24 (20.4)	
18	22.4-26.6	-	-	0.16 (22.5)	
18	40.2-49.8	-	-	0.22 (46.6)	0.13 (46.9)
20	22.6-29.9	0.25 (25.4)	-	-	-
20	42.2-58.7	-	-	0.23 (55.2)	-
21	0.8-9.0	-	-	0.12 (8.3)	-
21	25.2-33.6	-	-	0.14 (30.0)	-
24	26.3-30.8	-	-	0.16 (30.1)	-
26	9.8.2-23.6	0.15 (20.3)	-	0.28 (17.4)	0.15 (16.9)
26	27.9-32.9	-	-	0.17 (30.2)	-

^1^Number of bulls totaled 1810: 736 from United States (US); 306 from Australia (AU); 768 from New Zealand (NZ).

^2^Number of cows totaled 1933: 1047 from US; 886 from AU.

^3^Chr refers to chromosome.

^4^The largest interval and maximum location are in Megabases based on build UMD 3.1 (http://bovinegenome.org/cgi-bin/gbrowse/bovine_UMD31/).

### Characterizing the change in autozygosity within each population

The change of locus autozygosity (*∆*ROH45) across time was modeled using logistic regression of autozygosity on year of birth, where there were at least 40 genotyped animals. Initially the analysis was conducted on both bulls and cows, but no regions were found to be significant for the cows, possibly due to the narrow range in birth year of cows (Table 1) in comparison to the bulls. Therefore only the bull results are presented.

Multiple regions have undergone changes in autozygosity across time for the US and AU bull population, although no regions were significant for NZ (Additional file 1: Figure S1). The US bull population had 3 regions that have undergone autozygosity change across time and they are located on BTA1 (49.0-54.1; 68.5-75.7 Mb) and BTA11 (38.0-45.8 Mb). The two regions located on BTA1 were also shown to have different ROH45 frequencies across populations. The AU bull population had 2 regions that have undergone autozygosity change across time and they are both located on BTA9 (44.9-51.6; 61.3-68.7 Mb). Figure 4 clearly displays that differences across the genomes in the frequency of ROH regions exist, although the exact mechanism by which these occur, such as selection or drift cannot be disentangled.

### Effect of regions of high autozygosity or large differences across populations on yield traits

Regions that had differential ROH45 frequencies across population, high ROH45 frequencies in common across all population, and regions that have undergone significant autozygosity change across time were further investigated (N = 4849 SNP) to determine if SNP within these regions are associated with traits of economic importance. Yield deviations (YD) for cows that were derived from standardized lactation milk, fat and protein yield were weighted according to Garrick et al. [28] and a single marker regression model on the subset of SNP was used to describe the association between a trait and SNP. Markers with *p*-*values* smaller than 0.001 were declared significant. The false discovery rate (FDR) was calculated for each trait according to Benjamini and Hochberg [29].

Multiple regions contained SNP that were associated with milk, fat and protein yield and the FDR for milk, fat and protein yield was 0.30, 0.17, and 0.60, respectively. The region with the largest number of SNP was on BTA7 (38.6 – 58.0 Mb) and included 17 SNP associated with fat yield. Furthermore, a region on BTA17 (16.4 – 18.9 Mb) had 5 SNP associated with fat yield and a region on BTA3 had 6 SNP associated with milk yield. A complete list of the regions in addition to candidate genes are presented in Additional file 2: Table S2. A gene network analysis revealed a network involved in immune system function for milk yield (FDR = 12.7 percent) involving 11 genes that are outlined in Additional file 3: Figure S2 with 6 genes below the 0.001 threshold on BTA2 (*LCK*), BTA7 (*IL3*; *IL4*; *MKNK2; CSF2*) and BTA18 (*CEBPG*) and the remaining 5 below the 0.01 threshold.

## Discussion

The current study characterized the frequency and distribution of ROH across cow and bull populations derived from US, AU and NZ. Previous reports across multiple dairy breeds have similarly found that the NZ population is genetically different from other dairy cattle populations [18-21]. The correlations published by Interbull for milk, fat and protein yield [16] for the Jersey breed between US and NZ is weaker, i.e. further away from 1, in comparison to AU and NZ. As the genetic correlation deviates from 1 it indicates that the expression of the trait is different across environments [30]. A traditional method to examine the degree of differentiation is to compute Wright’s F_st_ statistic across two populations. The use of this measure is advantageous when large differences in allele frequencies occur, such as across cattle breeds. Within a breed, small differences in allele frequencies are expected across populations and particularly when there is some degree of genetic exchange, as is the case of the Jersey population. Due to this the usefulness of F_st_ to determine regions that are different within a breed is reduced, therefore alternative methods were used.

One such alternative method to characterize the genomic differences across populations is to compute the average or a specific region’s ROH frequency. The ROH metric has previously been used to examine population history [31] and as an alternative inbreeding metric [32,33]. Recently, Kim et al. [8] characterized the variation in ROH frequency in US Holstein dairy cattle utilizing an unselected Holstein population compared to two heavily selected Holstein populations. The mean number of ROH per individual was significantly lower in the unselected population than the two selected populations [8]. This study confirms that there are also differences in ROH levels across populations within the same breed, which may be due to different selection intensities across countries or different thresholds on the levels of allowable consanguineous matings.

Furthermore, Kim et al. [8] found that several of the regions that had differing levels of ROH across populations were associated with economically important traits including milk, fat and protein yield. The same approach was utilized in this study to detect signatures of selection in common and different across populations. Two regions on BTA3 and BTA7 were found to have high ROH45 frequencies across all populations. Previous studies have also found selection signatures on the same region of BTA3 [7,9], which contains the *SLC35A3* gene at 43.4 Mb. A mutation in this gene is known to cause a lethal recessive mutation in Holstein dairy cattle known as complex vertebral malformations (CVM) [34]. A lethal mutation would not give rise to the high level of autozygosity surrounding the CVM mutation, although selection at a nearby linked locus could potentially cause the region to have high levels of autozygosity. The selection signature on BTA7 confirms the findings of Kemper et al. in several cattle breeds [8] and Qanbari et al. in Fleckvieh cattle [35] and harbors multiple olfactory genes. Olfactory receptors detect and identify a wide range of odors, providing a cue for the animal to interact with its environment. Furthermore, gene duplications within the beef cattle genome tend to encode genes that interface with the external environment such as olfactory receptors [36], suggesting that they may be under strong selection for newly evolving functions.

Multiple regions of the genome displayed different autozygosity and interestingly, regions that were different across the US and NZ bull populations are similar to the results described by Kim et al. [8] where the comparison was between selected versus unselected Holstein populations. The regions include BTA1 (48.9-55.4 Mb), BTA2 (119.2-129.4 Mb), BTA9 (57.0-74.3 Mb), BTA14 (72.4-79.8 Mb), BTA16 (51.9-60.0 Mb) and BTA21 (25.2-33.6 Mb). This suggests that selection for yield has resulted in similar regions of high ROH45 frequency across different breeds.

Furthermore, SNP within regions that have undergone a significant autozygosity change have previously been reported to be associated with milk yield traits. The SNP with the largest significance within BTA9 (44.9-51.6) and BTA1 (49.0-54.1; 68.5-75.7) were within 1 Mb of SNP that have been previously shown to be associated with milk, fat and protein yield and fat and protein percentage, respectively [37]. It is unsurprising that no regions have undergone a significant autozygosity change in the NZ population, given their rather low and relatively uniform level of autozygosity across the genome in comparison to the greater variability in length and location of ROH in AU and US.

Functional analysis of genes within 500 kb in both directions of the significant SNP revealed regions involved in behavior (*NBEA*), milk fat synthesis (*FABP3*), fatty acid metabolism (*ACSL6*), and metabolism (*KCTD15*)*.* A previous study that investigated selection signatures across multiple beef and dairy breeds found a sweep region on BTA12 containing *NBEA* [38], which could be associated with traits associated with behavior [39]. Fatty acid binding proteins such as *FABP3* are one of the key intracellular FA transporters and is highly expressed in the mammary gland [40]. In general the favorable allele that was associated with the yield trait based on estimated SNP effects from a single marker regression model using the current dataset had a higher frequency in the US population in comparison to AU or NZ, which has lower levels of milk production, although other reasons may have caused allele frequencies to drift other than solely selection such as random genetic drift.

The gene network involving immune function is unsurprising due to a strong selection emphasis towards traits involving milk production which has led to a more pronounced negative relationship with metabolic, reproduction and health fitness traits [41]. In a study by Parker-Gaddis et al. [42] using US Holstein data, the genetic correlation for fitness traits such as ketosis, lameness, mastitis, metritis, and retained placenta were all negative with the US net merit index [43]. Furthermore, the particular environment that an animal is managed in may differentially compromise the host immunity and increase the incidence of variety of diseases [44,45] thereby augmenting the selection pressure on a given region. For the genes involved in immune function the frequency of the favorable allele based on estimated SNP effects from a single marker regression model using the current dataset was not consistently higher in a particular population.

Combining SNP assay data across multiple countries was initially aimed at increasing the reliability of genomic breeding value estimates [1,2]. Nonetheless, other potential uses can be garnered from the multi-country collaboration. One example, may be to use this *a priori* knowledge of the location of these genomic differences in mating schemes in order to decrease the level of homozygosity in the progeny at the genomic level. The availability of a multi-country reference population allows for the detection of a diverse set of haplotypes, which could potentially be exploited using methods such as optimum-contribution selection methodologies [46,47] that weights selection response versus future inbreeding. Furthermore, a multi-country reference population increases the likelihood of detecting selection candidates with favorable but different combinations of chromosomal segments [48]. Relationship matrices that characterize the similarity of haplotype segments [49] may allow for a more effective progeny inbreeding penalty. A sizable body of literature exists on using genomic information to constrain parental relationships and control the rate of inbreeding or level of homozygosity [50-54]. In general these methods constrain relationships averaged across the genome, although Pryce et al. [55] confirmed that certain regions have a larger impact on inbreeding depression than other regions. Therefore, optimum-contribution selection algorithms that incorporate this *a priori* knowledge of regions that have a large impact on inbreeding depression and different levels of ROH across countries, may be more effective in controlling homozygosity at the genomic level and minimizing inbreeding depression. In order for genomic information to be utilized in mating designs whole herd genotyping is required, which currently is not a common practice. As the technology improves and the cost of low-density genotyping platforms decreases, mating designs that utilize genomic information could assist producers in managing their herd at the genomic level.

Some limitations of the current study regarding ROH distribution and frequency could stem from the MAF threshold chosen, that may have resulted in removing SNP that have a low MAF in one population and higher MAF in another population, or SNP near fixation in all populations. As a consequence, potential regions that are similar and different across populations may not be detected if they are near fixation in one or more populations. This editing procedure was used because the detection of “hard sweep” selection signatures involving breed defining traits such as coat color or polledness was not the primary emphasis in this study. Also, it has been shown that the medium density SNP panel is not sensitive enough for the precise determination of short ROH segments [32]. A high density SNP panel was not available across all populations, but we anticipate that denser panels (or sequence data) should help to disentangle the selective history for short segments. Lastly, criteria used for inclusion of individuals in the genotyped populations may not be similar across populations, which may have resulted in genotyped animals in some of the populations not necessarily being representative of the animals within the given country. Multiple editing procedures were here used to minimize this phenomenon in order to make comparisons meaningful.

## Conclusions

Regions that displayed differential ROH45 frequencies across bull and cow resource populations from US, AU and NZ were characterized and the largest difference was between the US and NZ population which was in line with the PCA analysis. Regions of the genome that had high levels of autozygosity across all populations were found on BTA3 and BTA7. Furthermore a proportion of the regions that were different across populations were associated with milk yield traits. These subtle populations differences could potentially be exploited at the animal level in order to design mating schemes, that are tailored toward maximizing the level of heterozygosity along with superior additive genetics in the progeny, which will be the focus of future research.

## Methods

### Animal and genotypes

No animal care approval was required for the present manuscript because all records came from field data. The US resource population utilized in the study included genotypes obtained from the American Jersey Cattle Association while the AU and NZ resource population was provided by the Australian Dairy Herd Improvement Scheme (ADHIS; Melbourne, Australia). The majority of the US cows (n = 7458) were genotyped with a low density chip, either GGP (GeneSeek, Lincoln, NE), BovineLD (Illumina, San Diego, CA) or Bovine3K (Illumina, San Diego, CA)) and imputed to medium density (n = 61,013 SNP). The remaining cows (n = 777) were genotyped using the Illumina BovineSNP50 BeadChip (Illumina, San Diego, CA). The AU cows (n = 4075) were part of the Australian genomic reference population and were genotyped by the Dairy Futures CRC (Melbourne, Australia) with the Illumina BovineSNP50 BeadChip (Illumina, San Diego, CA). Bull genotypes from the Illumina BovineSNP50 BeadChip (Illumina, San Diego, CA) were also obtained from the American Jersey Cattle Association (n = 2394) and the Australian Dairy Herd Improvement Scheme (AU = 1069 bulls; NZ = 1748 bulls). The NZ population comprised of bulls genotyped by Livestock Improvement Corporation (Hamilton, New Zealand).

Genotype quality control, imputation and phasing were done within each population. For the US population genotype quality control included removing animals that had less than 90% of the SNP called, SNP with a minor allele frequency (MAF) below 0.01 or a p-value of a chi-square test for Hardy-Weinberg equilibrium less than 0.001. Full details of the quality control methods for the AU and NZ populations are described in detail in [56] and are similar to the rules applied to the US populations. The SNP unmapped to the Bovine Genome Build 4.0 (http://bovinegenome.org/cgi-bin/gbrowse/bovine_UMD31/) and SNP on sex chromosomes were excluded from the analysis. Missing SNP within the USA population were imputed using Beagle [57] and SNP with an imputation accuracy of less than 97.5% were removed. We recognize that a MAF threshold may result in removing SNP that have a low MAF in one population and higher MAF in the other population or SNP near fixation in all populations, nonetheless imputation accuracy was greatly impacted by MAF. The remaining SNP that passed quality control for the cow and bull groups were then combined resulting in 31,431 and 27,927 SNP in common between the groups, respectively.

In order to make comparisons across populations as equitable as possible a subset of the complete set of genotypes that met certain criteria were used to characterize difference across populations. To minimize the possible time trend effects and selective genotyping in a particular population cows and bulls included were selected that were born within a similar time frame. For the cow analysis, animals born within a three-year (2007–2009) period were used to make comparisons across and within populations. The AU cow resource population was created by selecting animals that had a large amount of individual phenotypic data and is tailored to represent the diversity across the AU Jersey population. To eliminate herds from the US that genotyped only a few of their elite cows, only herds that had greater than 20 genotyped individuals within a given year were used. The US animals (n = 1047) selected for the comparison came from herds that had genotyped on average 31 animals per year while all AU cows (n = 886) were used within the given years. For the bull analysis, animals born within a six-year period (2001–2006) were used. No criterion was used for the bulls on the number of genotyped animals within a year and herd, as the bulls were representative of the progeny tested bulls in each population. The total number of bulls was 306, 736, 768 for AU, US and NZ bulls respectively. The use of the same year classes in the analysis across bulls and cows was not possible due to fewer number of genotyped animals within a given year for the bulls in comparison to the cows. The number of animals by year class is outlined in Table 1.

### Principal component analysis

The SNP in common across the 2 cow (SNP = 31,431) and 3 bull (SNP = 27,927) populations were used to construct a GRM using the method outlined by Yang et al. [25]. Only cows and bulls born between 2007 to 2009 and 2001 to 2006, respectively, were used to construct GRM. Briefly, the GRM was calculated using:

$$ \frac{1}{N}{\displaystyle {\sum}_m\frac{x_m^2-\left(1+2{p}_m\right){x}_m+2{p}_m^2}{2{p}_m\left(1-{p}_m\right)}}, $$

where N is the number of SNP, p_m_ is the allele frequency of SNP_m_ and x_m_ is the genotype at SNP_m_. A PCA was conducted on the GRM matrix using the R function *eigen* [58]. The resulting matrix is a matrix of eigenvectors, referred to as principle components (PC), ordered by descending eigenvalues, where PC1 had the largest eigenvalue. The first two PC were plotted and annotated by country to determine the degree of genetic differentiation across the populations and the variance explained by the PC1 was calculated as the variance attributed to PC1 divided by the total variance.

### Characterizing the homozygosity across and within populations

Cows and bulls born between 2007 to 2009 and 2001 to 2006, respectively, were used to characterize the homozygosity across and within populations. Homozygosity characteristics for each population were measured as the overall genomic homozygosity (proportion of SNP that were homozygous across the entire genome) as well as the proportion of genome contained within a ROH. Using a sliding window approach with a fixed SNP length, a ROH was declared when a set number of contiguous homozygous SNP with no heterozygotes was observed. The sliding window approach started with the first SNP on a chromosome and combined all SNP within a given SNP number into a window, then the window was shifted by one SNP to form a new window and this process was repeated until the end of a chromosome. The SNP lengths considered were 45 (average ± SD = 3.44 ± 0.92 Mb), 70 (average ± SD = 5.45 ± 1.25 Mb) and 95 (average ± SD =7.47 ± 1.54 Mb). These SNP lengths were chosen to provide a range of ROH lengths. A minimum heterozygous threshold was not utilized here as it has been shown that setting a threshold for the number of heterozygous SNP within a ROH region potentially leads to inaccurate ROH calling at the boundaries of a ROH region [40]. The proportion of the genome contained with an ROH was estimated by the sum of ROH lengths (Mb) of an individual divided by the total Mb length across all 29 autosomes [8].

Differences across the genome in the location and length of stretches of homozygosity were investigated utilizing the method outlined by Kim et al. [8]. Briefly, the ROH45status of a SNP was defined based on whether it belonged to a ROH of at least 45 SNP. The ROH45 of a SNP was tagged as 1 if the SNP was in a ROH and 0 otherwise. A length of 45 was chosen for the ROH45 metric based on the average Mb length of 3.44 and a previous study has used a similar SNP length value [8]. The ROH45 metric is advantageous compared to the conventional ROH since it is able to capture the number of times a SNP is in a ROH without declaring the beginning and end of a ROH. The ROH45 value for each SNP was compared across the two populations using a chi-square test with 1 degree of freedom. A statistical threshold was determined using a permutation test (n= 1,000 samples) [27]. Briefly, within each analysis the populations were combined and animals were randomly allocated into groups that were the same size as the original data (n=2 for cows, n=3 for bulls). The ROH45 value for each SNP by group was calculated and significance was reported as the number of times the observed difference was greater than the permutation sample difference across all SNP. The presence of differential autozygous regions was declared as contiguous significant differences (P < 0.001) of at least 45 SNP for regions greater than 4 Mb in length. The presence of regions with high levels autozygosity in common across all populations was declared as contiguous SNP within the top 2.5% of at least 45 SNP and greater than 4 Mb in length.

### Change of autozygosity across time

The change of locus autozygosity (*∆*ROH45) across time was modeled using logistic regression as described by Kim et al. [8]. US cows born after 2002, AU cows born between 1999 and 2008 and bulls born between 1990 and 2010 for AU, NZ and US were utilized in the study to ensure a reasonable size for each individuals/year class. Briefly, the logistic regression model was:

$$ {F}_l=\frac{e^{\left(\alpha +\beta YB\right)}}{1+{e}^{\left(\alpha +\beta YB\right)}} $$

Where ROH45 is the autozygous status of a locus (0, 1), YB is the year of birth of an individual, *α* is the intercept of the model and *β* is the change of annual locus autozygosity. Statistical thresholds were determined using a permutation test [27] (n = 1,000 samples) similar to the one previously discussed. The presence of autozygosity change across time was again declared for contiguous significant differences (P < 0.001) of at least 45 SNP in regions of at least 4 Mb.

### Effect of regions of high autozygosity or large differences across populations on yield traits

Regions that were declared significantly different or similar and regions that have undergone significant autozygosity change across time were investigated to determine if SNP within these regions are associated with yield traits. Phenotypic information was only available for the cows (AU: n = 3974 animals; US: n= 6750 animals) and included standardized lactation milk, fat and protein yield. Yield deviations were calculated separately for each population, by adjusting for the following effects using ASReml [59] in each population:

$$ {y}_{ijklm}=\mu +HY{S}_i+ parit{y}_j+ mont{h}_k+ age+{e}_{ijklm}, $$

where y_ijklm_ refers to either standardized milk, fat or protein yield, *μ* is the intercept, HYS_i_ is the fixed effect of herd-year-season of calving, parity_j_ was the fixed effect of parity, month_k_ was the fixed effect of month of calving, and age was the regression of age at first calf. Random effects included the residual. For cows with multiple lactation records, mean adjusted records from the above model were used in the analyses as yield deviations. Yield deviation of cows were standardized to have a mean of 0 and a variance of 1 to make variances similar across populations. Then the following single marker regression model was used:

$$ {y}_{ijk}=\mu +po{p}_i+SN{P}_j+{u}_k+\frac{e_{ijk}}{w_{ijk}} $$

where y_ijk_ refers to the yield deviation for milk, fat or protein yield, *μ* is the intercept, pop_i_ is the fixed effect of country of origin, SNP_j_ is the additive effect of SNP. Random effects included u_k_ the additive genetic effect of the k^th^ individual assumed ~ N(0, **A**${\sigma}_u^2$), with **A** representing the additive relationship matrix derived from a pedigree that traced back at least 4 generations. The random residual, e_ijk_, was weighted by w_ijk_ for the k^th^ individual according to Garrick et al. [28]. The formula used to calculate w_ijk_ was:

$$ \frac{\left(1-{h}^2\right)}{h^2+\frac{1+{r}^2\left(l-1\right)}{l}-{h}^2}, $$

where *h*^*2*^ refers to the heritability, *r*^*2*^ refers to the repeatability and *l* refers to the parity. The values used for h^2^ and r^2^ were 0.25 and 0.43 and were averages across all three traits. The p-values that were smaller than 0.001 were declared as significant and the false discover rates (FDR) were calculated according to Benjamini and Hochberg [29].

### Gene annotation

Cow positional candidate genes using *Bos Taurus* assembly (UMD3.1; Ensemble 68) with regions declared significantly different and similar across the populations were obtained for functional characterization and the identification of gene ontology terms using DAVID [60,61] and gene network work analysis using GeneMANIA [62]. Regions surrounding SNP associated with milk yield traits were extending 125 kb in both directions for characterization. Furthermore, previously identified QTL from CattleQTLdb [63] and a tabulated list of QTL for milk production and mastitis [64] were used to locate previously known QTL affecting traits of economic importance.

## Additional files

Additional file 1: Figure S1. Change in autozygosity across time for the bull populations^1^. ^1^AU = Australia (n = 889); US = United States (n = 1556); NZ = New Zealand (n=2131). ^2^ Dashed line represents significance threshold (P-value < 0.001). Additional file 2: Table S2. Regions of the genome associated with milk yield traits. ^1^Chr refers to chromosome. ^2^The largest interval and maximum location are in Megabases based on build UMD 3.1 (http://bovinegenome.org/cgi-bin/gbrowse/bovine_UMD31/). Additional file 3: Figure S2. Gene network for milk yield^1^. ^1^Genes related to immune function highlighted in red.
