# Supplementary figures and images for: Characterizing homozygosity across United States, New Zealand and Australian Jersey cow and bull populations
Source: BMC Genomics. 2015 Mar 15;16(1):187. doi: 10.1186/s12864-015-1352-4 (PMC4460752; doi:10.1186/s12864-015-1352-4)

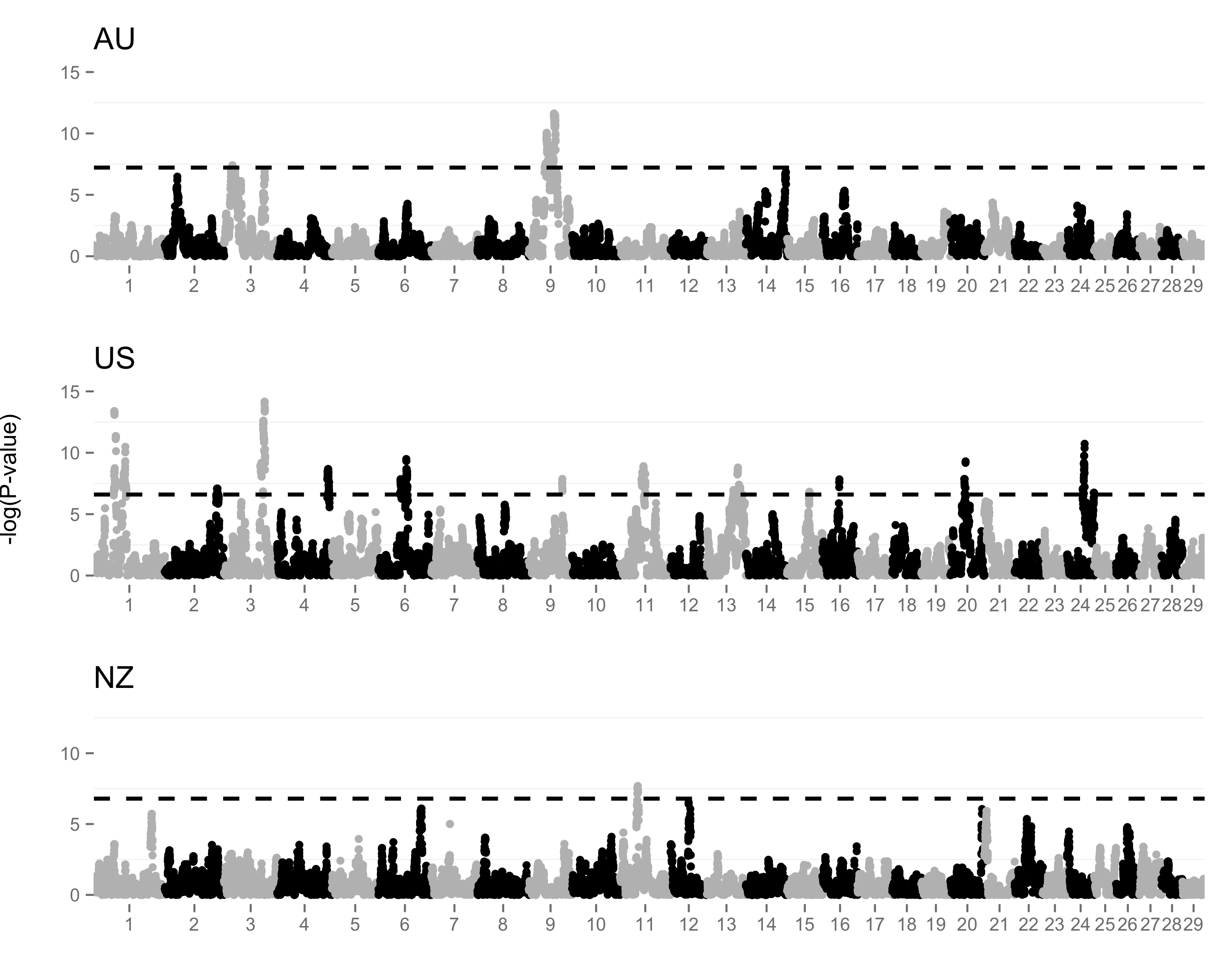

Supplement: Additional file 1: Figure S1. — Change in autozygosity across time for the bull populations1. 1AU = Australia (n = 889); US = United States (n = 1556); NZ = New Zealand (n=2131). 2 Dashed line represents significance threshold (P-value < 0.001). [file 12864_2015_1352_MOESM1_ESM.png]

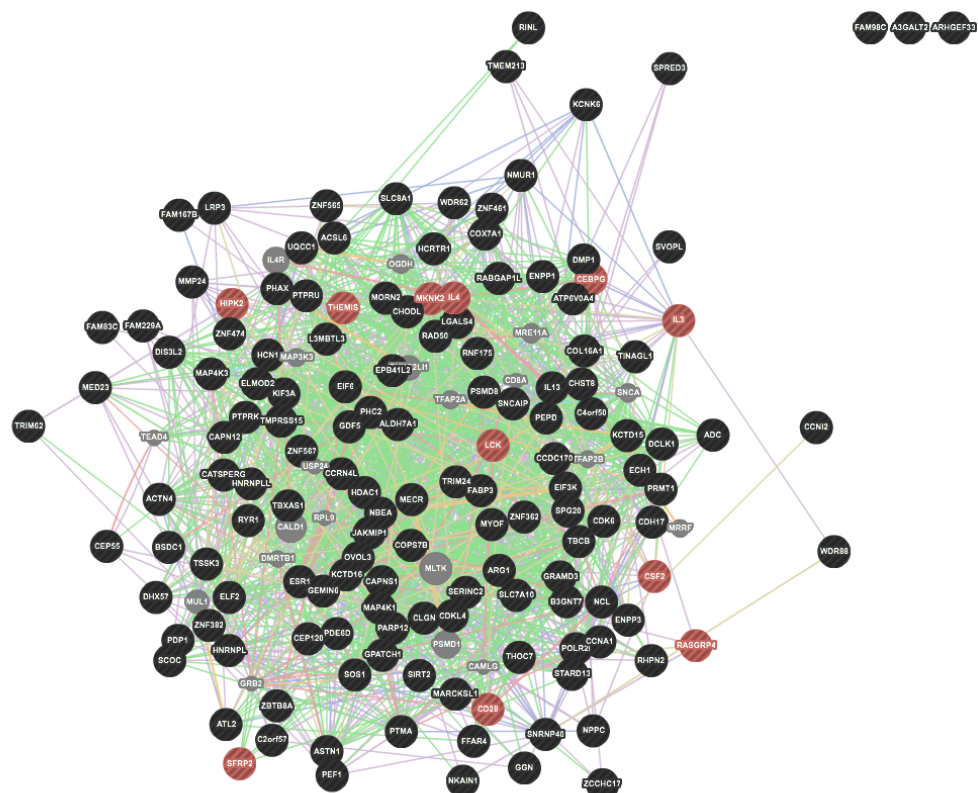

Supplement: Additional file 3: Figure S2. — Gene network for milk yield1. 1Genes related to immune function highlighted in red. [file 12864_2015_1352_MOESM3_ESM.pdf]
