# Supplementary material for: Characterizing homozygosity across United States, New Zealand and Australian Jersey cow and bull populations
Source: BMC Genomics. 2015 Mar 15;16(1):187. doi: 10.1186/s12864-015-1352-4 (PMC4460752; doi:10.1186/s12864-015-1352-4)
Supplement: Additional file 2: Table S2. — Regions of the genome associated with milk yield traits. 1Chr refers to chromosome. 2The largest interval and maximum location are in Megabases based on build UMD 3.1 (http://bovinegenome.org/cgi-bin/gbrowse/bovine_UMD31/). [file 12864_2015_1352_MOESM2_ESM.doc]

**Table S2.** Regions of the genome associated with milk yield traits.

| Chr^1^ | Trait | Region^1^ | Number of SNP | Location(s) of Associated SNP^2^ | Candidate Gene  (Function / Previously Associated Trait) | Average Difference Between Favorable Allele  (US – AU) |
| --- | --- | --- | --- | --- | --- | --- |
| 2 | Milk Yield | 119.2-129.4 | 6 | 120.1 – 122.7 | *FABP3* (Milk Fat Synthesis)  *LCK* (Immune System) | -0.036 |
| 6 | Milk Yield | 102.2-106.2 | 1 | 105.0 |  | 0.069 |
| 7 | Milk Yield | 19.9-34.6 | 1 | 23.8 | *ACSL6* (Fatty Acid Metabolism)  *MKNK2; IL4; IL3; Csf2* (Immune System) | 0.053 |
| 12 | Milk Yield | 22.9-29.4 | 2 | 25.7-25.8 | *NBEA* (Psychiatric Disorders) | 0.040 |
| 17 | Milk Yield | 12.6-25.2 | 1 | 18.9 | *ELF2* (Milk Yield) | 0.094 |
| 18 | Milk Yield | 40.2-49.8 | 2 | 44.3 | *KCTD15* (Metabolism)  *RASGRP4* (Immune System) | 0.004 |
| 4 | Fat Yield | 8.0-12.2 | 2 | 9.6-11.0 | COL1A2 (Fat Yield) | -0.054 |
| 7 | Fat Yield | 19.9-34.6 | 2 | 22.9-23.0 | *ACSL6* (Fatty Acid Metabolism)  *MKNK2; IL4; IL3; Csf2* (Immune System) | 0.012 |
| 7 | Fat Yield | 35.6 to 58.4 | 17 | 38.6 – 58.0 | Multiple Olfactory Receptors | 0.009 |
| 17 | Fat Yield | 12.6-25.2 | 5 | 16.4-18.9 | *ELF2* (Milk Yield) | 0.060 |
| 4 | Protein Yield | 102.2-106.2 | 1 | 103.1 |  | -0.008 |
| 7 | Protein Yield | 49.0-58.4 | 1 | 52.4 | Multiple Olfactory Receptors | 0.026 |
| 12 | Protein Yield | 22.9-29.4 | 1 | 25.8 | *NBEA* (Psychiatric Disorders) | *0.038* |
| 17 | Protein Yield | 12.6-25.2 | 2 | 17.6 | *ELF2* (Milk Yield) | *0.031* |
| 18 | Protein Yield | 40.2-49.8 | 2 | 44.0-44.3 | *KCTD15* (Metabolism)  *CEBPG* (Immune System) | *0.000* |

^1^ Chr refers to chromosome.

^2^ The largest interval and maximum location are in Megabases based on build UMD 3.1 (http://bovinegenome.org/cgi-bin/gbrowse/bovine_UMD31/).
